# Intraocular pressure control after implantation of experimental antiglaucoma shunt versus trabeculectomy

**DOI:** 10.22336/rjo.2026.10

**Published:** 2026

**Authors:** Maria Iacubitchii, Ala Paduca, Oleg Arnaut, Eugeniu Bendelic

**Affiliations:** 1 Department of Ophthalmology, “Nicolae Testemitanu” State University of Medicine and Pharmacy, Chişinău, Republic of Moldova; 2 Department of Human Physiology and Biophysics, “Nicolae Testemitanu” State University of Medicine and Pharmacy, Chişinău, Republic of Moldova

**Keywords:** glaucoma, antiglaucoma shunt with valve, intraocular pressure, IOP = intraocular pressure, PMMA = polymethyl methacrylate

## Abstract

**Introduction:**

Glaucoma treatment includes various methods, such as eye drops, laser treatment, and surgery. The known complications of trabeculectomy have motivated the development of alternative filtering devices, including an antiglaucoma shunt with a valve.

The research aims to assess the efficacy of the antiglaucoma shunt in rabbits with induced hypertension.

**Materials and methods:**

The preclinical study compared intraocular pressure (IOP) values in 42 rabbits’ eyes with induced steroid-induced intraocular hypertension that underwent trabeculectomy (Group A, n=21) versus antiglaucoma shunt implantation (Group B, n=21). Statistical analysis used a linear mixed model, with model validation through residual diagnostics, Q-Q plots, and evaluation of random effects.

**Results:**

Both surgical methods produced a significant and sustained reduction in IOP compared with induced ocular hypertension values (average decrease ≈ of 6,5 mmHg per time unit, p < 0,001). Group A and B did not differ statistically in their ability to lower IOP at any period of examination (baseline: p=0.91; induced hypertension: p=0.92; 1 month postoperative: p=0.11; and 3 months postoperative: p=0.18). There was no systemic group bias, but random effects analysis indicated moderate inter-subject variability.

**Discussion:**

The results demonstrate that in a steroid-induced rabbit model of intraocular hypertension, both the new design shunt and conventional trabeculectomy offer efficient and sustained IOP reduction. Across all time points, the absence of significant differences between the groups indicates that the antiglaucoma shunt performs comparably to the established surgical technique.

**Conclusions:**

The implantation of the antiglaucoma shunt with valve showed IOP-lowering efficacy comparable to that of traditional trabeculectomy, maintaining sustained pressure decrease over the 3-month follow-up. According to these findings, the antiglaucoma shunt may be a viable surgical alternative for glaucoma treatment, warranting further research through long-term animal studies and clinical trials. It offers new possibilities in glaucoma treatment, with a postoperative IOP decrease of 35% or more.

## Introduction

Glaucoma is a complex eye pathology that affects the optic nerve and leads to specific functional deficits. It represents the second leading cause of blindness globally, according to the World Health Organization [1-3]. In 2020, glaucoma caused roughly 4.14 million individuals to become visually impaired and 3.61 million people to become blind worldwide [4]. Globally, an estimated 64.3 million persons between the ages of 40 and 80 had glaucoma in 2013, rising to 76.0 million in 2020 and 111.8 million in 2040 [5]. Decreased visual acuity due to the chronic nature of glaucoma disease was highly associated with worse quality of life and greater levels of anxiety and depression [6]. The cost for the treatment is impacted by the asymptomatic nature of the pathology, lack of public knowledge and education, a lack of health care, noncompliance with recommended therapy, and inadequate treatment.

Nowadays, the surgical approach to treating glaucoma has gained popularity and is frequently the preferred treatment even when it is initially diagnosed. At the moment, the drainage surgery for glaucoma is given particular consideration. There is a variety of filtering devices, but there is no solid evidence supporting the superiority of any model [7,8]. Drainage surgery can have certain disadvantages, though. Numerous studies discuss the characteristics and complications that can appear after implantation of these devices [9,10]. It is important to maintain the proper position of the antiglaucoma filtering shunts in the anterior chamber, avoiding the cornea and uveal structures, which can lead to decompensation of the corneal endothelium [11].

In addition, it is important that the design of the glaucoma devices, as a heavy one, is prone to perforation. The materials used also play an important role, as they can cause oxidation or activate local ocular reactivity with device occlusion [12]. Reimplantation of a shunt in cases of inadequate positioning is difficult, traumatic, and requires specialized surgical techniques [13,14]. New technologies are developed through efforts to address these issues.

The study aimed to evaluate the efficacy of the polymethyl methacrylate (PMMA) antiglaucoma shunt with an original valve design in controlling intraocular pressure (IOP) in a model of induced ocular hypertension.

## Materials and methods

### Design of the antiglaucoma shunt

**Fig. 1:**
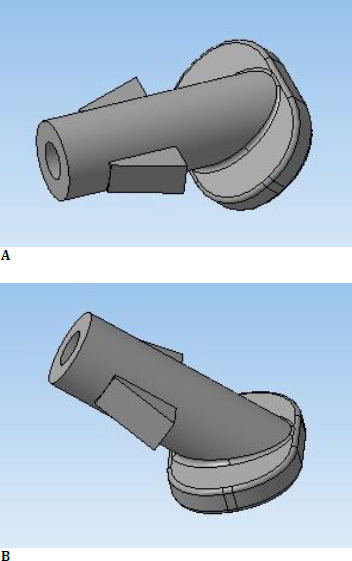
3D design of the antiglaucoma shunt with valve superior (**A**) and lateral view (**B**) [16]

**Fig. 2:**
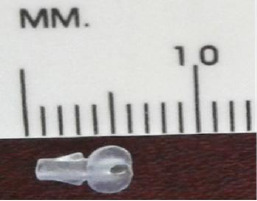
Final form of the antiglaucoma shunt with valve [16]

The antiglaucoma shunt with valve is made of PMMA and was developed within the Department of Ophthalmology at “Nicolae Testemitanu” State University of Medicine and Pharmacy (**Fig. 1A, B, 2**) [15,16].

Shunt implantation will not cause additional stress, such as pressure-related injury to the surrounding ocular tissues, due to its distinctive structure. Also, the valve inside the filtering device will allow drainage of excess aqueous humor if IOP increases.

As a material for the anti-glaucoma shunt with valve, PMMA was selected due to its high biocompatibility. PMMA offers numerous benefits, including excellent tolerance for ocular tissue, a lower inflammatory response to foreign bodies, higher uveal biocompatibility, light weight, and stability [17]. PMMA’s hydrophobic properties after the heparin-coated surface procedure are known to cause minimal inflammatory response, reducing the risk of drainage blockage [18].

### Experimental design

Forty-two white New Zealand rabbits, aged 3 ± 0,5 months, weighing 3,2-3,4 kg, were used in the preclinical study. All animals were treated in accordance with the guidelines approved by the Research Ethics Committee of “Nicolae Testemiţanu” State University of Medicine and Pharmacy, Chişinău, Republic of Moldova (no. 20 from 21.11.2017).

Each New Zealand rabbit received a weekly local subconjunctival injection of 0,7 ml of betamethasone suspension in one eye (right eye) to induce ocular hypertension, according to protocols developed by Hester D. E. and Khan S.U. [19-21]. For three weeks, the procedure was carried out under local anesthesia. The IOP before induction and the hypertension obtained were measured using a Tono-PenXL (Reichert, SUA). The IOP was measured five times, and the tonometer reported the mean IOP.

The rabbits were randomly divided into two groups: Group A underwent trabeculectomy (n=21), and Group B underwent implantation of an antiglaucoma shunt (n=21). A single surgeon conducted the filtration surgery. The procedure was performed under aseptic conditions with local (proparacaine hydrochloride 0,5%) and general anesthesia (intramuscular injection of xylazine 5 mg/kg and ketamine 35 mg/kg).

A routine trabeculectomy surgery was performed on each rabbit from Group A. After the conjunctival incision, a 2/3 thickness scleral flap was performed and extended into the transparent cornea and dissected anteriorly to the limbus. A peripheral iridectomy was carried out after the trabeculectomy. The scleral flap was sutured with 10-0 nylon, and the conjunctiva was closed with an 8-0 nylon.

Group B underwent implantation of an antiglaucoma shunt with a valve. A conjunctival flap based on the fornix was used in each case. A 2/3 thickness scleral flap was performed at the 12 o’clock position. At the base of the flap, an incision of 1,5 mm was made with implantation of the shunt, ensuring its proper positioning in the anterior chamber, with no contact with the iris surface or cornea. The sclera was closed with 10-0 nylon, and the conjunctiva was sutured with 8-0 nylon [22,23].

Following surgery, both groups received topical antibiotic and corticosteroid therapy in the form of OD subconjunctival injections of dexamethasone 4 mg/1 ml, 0,3 ml, and gentamicin sulfate 80 mg/2 ml, 0,3 ml, and tobramycin ointment 0,3%. Additionally, the same local antibacterial and anti-inflammatory therapy was administered to Group A and Group B.

In the 1st and 3rd months after the surgical procedure was performed, examination was performed using the portable slit lamp microscope (SN2108113H, China) and tonometry. Under biomicroscopy, the filtering bleb, cornea, anterior chamber, lens, and anterior vitreous body were examined.

The statistical analysis was conducted using a linear mixed model. To ensure the robustness of the results, model validation was performed through residual diagnostics and Q-Q plots. Additional checks included posterior predictive assessment and the evaluation of random effects.

## Results

**Fig. 3:**
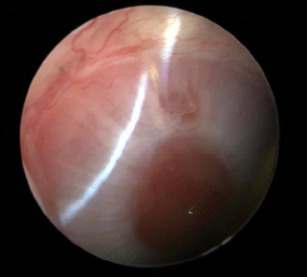
The anterior segment of the eye from the Group B at the 3^rd^ month post-surgery

Post-surgery, the rabbits in both groups exhibited typical feeding, hydration, and excretion behaviors. Ophthalmological investigation revealed no visible conjunctival congestion or a normally functioning filtration bleb. The cornea was free of oedema or precipitates, with no inflammation in the anterior chamber, and the lens and vitreous were transparent (**Fig. 3**).

Among the early complications observed within the first 3 months in both study groups, one case (4,76%) was reported in the trabeculectomy group, represented by transient hyphema. It was identified as a mild deposition of red blood cells on the anterior lens capsule, which resolved with standard postoperative therapy without the need for further medical intervention.

The efficacy of the antiglaucoma shunt was evaluated compared with conventional trabeculectomy. The two groups of animals underwent IOP monitoring at several time points: baseline (initial IOP), after induction of intraocular hypertension, and at 1 and 3 months after surgery (**Table 1**).

Both groups showed a similar trend in IOP over time: a significant increase at induction, followed by a decrease at 1 month and a return to values near the initial level at 3 months. The baseline IOP for Group B ranged from 12 to 16 mmHg, with a mean of 13,8 ± 1,2 mmHg. During the induction stage, IOP increased to 28,2 ± 1,6 mmHg, then decreased to 22,4 ± 1,9 mmHg at 1 month and 15,1 ± 1,2 mmHg at 3 months. Similarly, for Group A, the baseline IOP was 13,8 ± 1,4 mmHg, and the IOP during induced ocular hypertension was 28,2 ± 1,5 mmHg. At the 1-month follow-up, a reduction of 21.5 ± 1.8 mmHg was observed, and at 3 months, 14.7 ± 1.1 mmHg.

**Table 1:** PIO values (mmHg) by group and evaluation period

Group	Group A				Group B			
	Baseline N=21^1^	Induced hypertension N=21^1^	1 month N=21^1^	3 months N=21^1^	Baseline N=21^1^	Induced hypertension N=21^1^	1 month N=21^1^	3 months N=21^1^
**IOP**	13,8 (1,4)	28,2 (1,5)	21,5 (1,8)	14,7 (1,1)	13,8 (1,2)	28,2 (1,6)	22,4 (1,9)	15,1 (1,2)
	14,0 (2,0)	28,0 (2,0)	21,0 (3,0)	14,0 (1,0)	14,0 (2,0)	28,0 (3,0)	22,0 (3,0)	15,0 (2,0)
	11,0 16,0	26,0 31,0	18,0 24,0	13,0 17,0	12,0 16,0	26,0 31,0	19,0 26,0	13,0 18,0

^1^ Mean (SD) Median (IQR) Min Max

To assess the influence of time and surgery type on IOP, a linear regression model was used. It included the following predictors: type of surgery (antiglaucoma shunt implantation, Group B – reference vs. trabeculectomy, Group A), time of assessment (Time), initial IOP value, and interaction between time and type of intervention (**Table 2**).

The results indicated a significant effect of time on IOP reduction (Estimate= -6,52, p < 0,001), suggesting an average reduction of approximately 6,5 mmHg per unit of time, regardless of the type of intervention. Also, higher initial IOP values were significantly associated with higher subsequent IOP values (Estimate = 0.583, p < 0.001), confirming the influence of baseline IOP on the IOP trajectory.

No significant differences were observed between groups at baseline (Estimate = 0.107, p = 0,852), supporting the homogeneity of the groups before surgery. Furthermore, the interaction between time and type of intervention was not significant (Estimate = -0.262, p = 0.278). This indicated that both surgical procedures caused a similar decrease in IOP over time.

**Table 2:** Linear mixed model results

Term	Estimate	p-value	Interpretation
Intercept	26,90	< 0,001***	Average IOP at baseline (Time=0, Intervention= reference group, and IOP initial=0)
Time	-6,53	< 0,001***	On average, IOP decreased by ~ 6,52 units per time unit across all groups, holding other variables constant
Intervention Group A	0,107	0,852	No significant difference at baseline between Group A and the reference (Group B)
Baseline IOP	0,583	< 0,001***	Higher initial values of IOP predicted higher current values - as expected
Time: Intervention Group A	-0,262	0.278	The effect of time did not significantly differ between intervention groups

### Model validation

The adequacy of the regression model was evaluated using a comprehensive set of diagnostic plots that assessed the key assumptions of mixed-effects linear regression.

By examining the graph of Pearson standardized residuals as a function of fitted values, the adequacy of the regression model was evaluated. Since there was no clear pattern or systematic relationship among the residuals, which were randomly dispersed about the zero line, the linear relationship between the predictors and the outcome variable was appropriately recorded. The relatively constant spread of the residuals across the full range of fitted values suggests the absence of heteroscedasticity, thus satisfying a key assumption of linear regression. Despite a few extreme values (residuals > ±2), they could be interpreted as isolated observations or normal biological variation, as they did not appear to affect the overall model structure significantly.

To assess the assumption of normality of residuals, a normal Q-Q (Quantile-Quantile) plot (**Fig. 5**) was created. The plot showed that the standardized residuals were roughly normally distributed, clustering near the reference line. Although there were slight variations at the extremes, especially in the distribution’s tail, they did not affect model validity. The model assumed normality, which supported statistical inference based on linear regression.

**Fig. 4:**
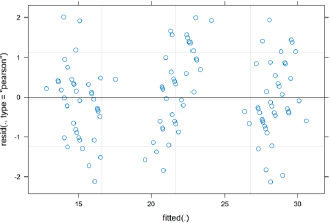
Predicted values of IOP

**Fig. 5:**
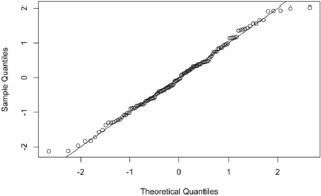
Normal Q-Q plot

**Fig. 6:**
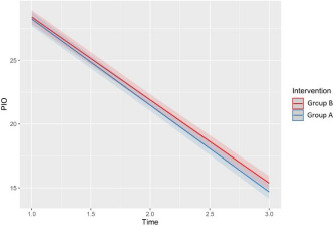
Predicted values of IOP

In the analysis of model-predicted IOP values over time (at 1 and 3 months postoperatively) in both groups, a clear reduction was observed (**Fig. 6**). The model showed a consistent and significant decrease in IOP over time. The regression model estimated a linear decline in IOP following surgery for Group B (red line) and Group A (blue line), with narrow confidence bands indicating high predictive accuracy.

Although Group B showed slightly higher predicted IOP values at each time point than Group A, the overlapping confidence intervals suggested this difference was not statistically significant.

**Fig. 7:**
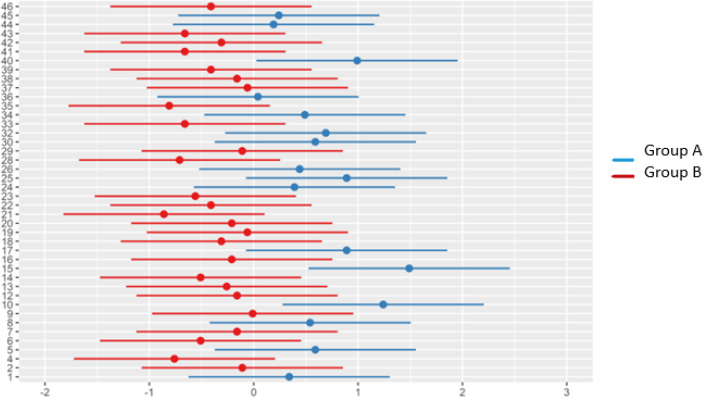
Random effects analysis

The distribution of random effects for every animal, stratified by intervention group (Group A- blue, Group B- red), showed moderate inter-individual variability, with estimated ranging between -2 and +2, without extremes that could suggest errors or strong isolated influences (**Fig. 7**). The symmetrical distribution around 0, which was expected for random effects confirmed that the model was not systematically affected by a particular subset of subjects.

The confidence intervals for the random effects were relatively balanced and of similar length across subjects, suggesting stable and robust estimates. No disproportionate influence on the model’s results was observed, as no subjects exhibited extreme values (> 3). Furthermore, the spread and central tendency of random effects were comparable across groups, suggesting similar variability patterns across treatments.

**Fig. 8:**
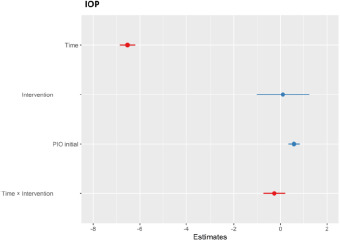
Forest plot of model coefficients (red line - statistically significant coefficients, blue line - statistically insignificant coefficients)

The calculated coefficients and 95% confidence intervals for the predictors in the mixed-effects model used to measure intraocular pressure are displayed in the forest plot (**Fig. 8**). Time showed a statistically significant negative coefficient, indicating that the IOP consistently decreased over time in both groups. This effect was robust, as the confidence interval did not cross zero. The intervention showed a small, positive, but non-significant coefficient, suggesting no difference in IOP between the control and reference groups. The initial IOP showed a positive association with subsequent IOP values, although this effect did not reach statistical significance in the model. The interaction term (Time x Intervention) showed a slightly negative coefficient, suggesting a slightly faster decrease in IOP for Group A than for Group B; however, the effect size was small and not statistically significant.

**Fig. 9:**
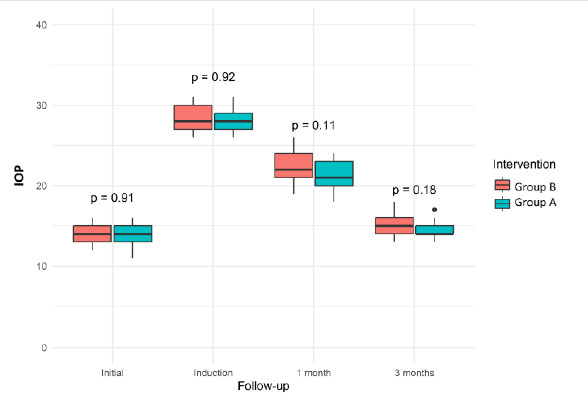
IOP changes in each group for the follow-up period

Box-plot analysis of IOP values in both groups was performed for all follow-up periods (initial, hypertension induction, 1 month, and 3 months after surgery) (**Fig. 9**). Baseline IOP values were almost identical between the groups, with no statistically significant difference (p=0.91). This fact confirmed the homogeneity before any intervention. IOP increased significantly and similarly in Groups A and B after induced hypertension (p=0.92), suggesting that the induction technique was successful and created similar preoperative conditions.

At 1 month, both groups showed a significant reduction in IOP compared to the induction phase. The mean for Group A appeared to be slightly lower than for Group B, but the difference was not statistically significant (p=0.11). IOP continued to decrease in both groups by 3 months postoperatively (p=0.18), with mean values similar across surgical methods, and no significant differences were observed. Although there were a few outlier values, they did not alter the overall trend.

## Discussion

Many important risks affect the success rate of glaucoma drainage surgery. Through these can be mentioned excessive fibrosis with bleb encapsulation, drainage blockage due to inflammation from aqueous humor [9,10], and corneal endothelial decompensation [24]. Endophthalmitis and erosion may result from devices placed in the subconjunctival space. These complications might require the removal of the glaucoma drainage devices, which could lead to glaucoma surgery failing.

Trabeculectomy is a classic surgical procedure used to lower the IOP; nevertheless, it is often required to be used in conjunction with local hypotensive medication. Also, a high percentage (30-50%) of interventions fail because of fibrosis [25].

All of these points require more research on glaucoma surgical treatment. The development of new designs and biomaterials for glaucoma drainage devices is ongoing. New techniques are being developed in response to these important considerations to improve surgical outcomes and reduce complications.

The effectiveness of the polymethyl methacrylate (PMMA) antiglaucoma shunt was investigated in an in vivo study of induced ocular hypertension. Clinical and functional data from 21 operated eyes of 21 white New Zealand rabbits, aged 3 ± 0,5 months, weighing 3,2-3,4 kg, with an implanted antiglaucoma shunt, served as the basis for the experimental study (Group B). The control group consisted of 21 operated eyes of 21 rabbits with the same zoomorphic characteristics that had undergone trabeculectomy (Group A).

In our study, no symptoms of conjunctival congestion, corneal opacification, or visible anterior chamber inflammation were observed. The transparency of the crystalline lens and anterior vitreous body was noted. Since there were no ocular or systemic side effects from the antiglaucoma shunt, its effectiveness was evaluated over 3 months. Transient hyphema was noted in a single case (4,76%) within Group A, an early postoperative complication that resolved with routine medical therapy.

To evaluate the efficacy of the antiglaucoma shunt, the animals were monitored for intraocular pressure at 3 time points: baseline (IOP), after induction of intraocular hypertension, and 1 and 3 months after surgery. The baseline IOP was the same in both groups (13,8 ± 1,4 mmHg for Group A and 13,8 ± 1,2 mmHg for Group B), as at the hypertension stage: 28,2 ± 1,5 mmHg for Group A vs. 28,2 ± 1,6 mmHg for Group B. After the implantation of the antiglaucoma shunt, the IOP decreased to 22.4 ± 1,9 mmHg at 1 month and 15,1 ± 1,2 mmHg at 3 months. Similarly, for Group A, at a 1-month follow-up, a reduction to 21,5 ± 1,8 mmHg was observed, and at 3 months to 14,7 ± 1,1 mmHg. The absence of significant differences between baseline and induced ocular hypertension groups validated the randomization and homogeneity of the groups.

The findings were supported by robust statistical modeling and comprehensive diagnostics. Both surgery methods significantly and sustainably reduced IOP, and over time, there was no statistically significant difference between the groups. The average reduction of approximately 6,5 mmHg per unit of time (p<0,001) is consistent with scientific literature [26], which highlights the physiological tendency for postoperative pressure to stabilize. This effect can be explained by reduced postoperative inflammation, normalization of aqueous humor drainage [27], and progressive tissue adaptation to the new hydrodynamic equilibrium. Notably, there was no significant interaction between the time and intervention, suggesting that pressure reduction remained similar throughout the follow-up period. This outcome is valuable because it suggests that the tested antiglaucoma shunt may be an appropriate alternative to traditional surgery, especially when a stable reduction in IOP is desired. These results are consistent with research on glaucoma drainage implants, such as the Baerveldt and nano-structured shunts, which have shown durable reduction in IOP in rabbit models [28].

For both surgical approaches, the predicted IOP trajectories showed comparable decreases, with overlapping confidence ranges suggesting no variation in efficacy. These results were supported by box-plot comparisons at baseline, post-induction, 1, and 3 months postoperatively; both groups exhibited similar patterns, with an induced rise of IOP followed by gradual normalization (p values: 0,91, 0,92, 0,11, 0,18).

The similarity of the antiglaucoma shunt to trabeculectomy in terms of IOP reduction suggests it could be a good surgical alternative, given its favorable postoperative results, easier technique, and improved reproducibility. This is especially important in light of increased interest in alternative implant designs aimed at reducing complications such as hypotony, bleb fibrosis, and occlusion [9,10,12].

The study had several limitations, though. Although models of filtering device implantation and well-known techniques for inducing ocular hypertension have been modified to resemble the human glaucomatous process closely, the results may not always match expected findings in humans. Additionally, the short follow-up period did not allow identification of potential late consequences.

## Conclusions

In this experimental study, a newly designed antiglaucoma shunt with a valve was implanted in rabbit eyes, and no significant intraocular or systemic adverse effects were observed during the observation period. This suggests a favorable safety profile for the device. The antiglaucoma shunt reduced IOP to a level similar to that achieved with trabeculectomy, the current gold-standard surgical approach for treating open-angle glaucoma.

These preliminary findings support the potential of this novel shunt as an alternative surgical approach, combining effective IOP control with a potentially lower risk of postoperative complications. Further research is warranted to assess the long-term outcomes, biocompatibility, and suitability of this device for human application in glaucoma surgery.

## Authorization for the use of human and animal subjects

Ethical approval: The research involving animal subjects complies with all relevant national regulations and institutional policies, and has been approved by the Research Ethics Committee of “Nicolae Testemiţanu” State University of Medicine and Pharmacy, Chişinău, Republic of Moldova (no. 20 from 21.11.2017).

## References

[1] Cook C, Foster P (2012). Epidemiology of glaucoma: what’s new?. Can J Ophthalmol.

[2] Quigley H, Broman AT (2006). The number of people with glaucoma worldwide in 2010 and 2020. Br J Ophthalmol.

[3] Allison K, Patel D, Alabi O (2020). Epidemiology of Glaucoma: The Past, Present, and Predictions for the Future. Cureus.

[4] Bourne RRA, Jonas JB, Friedman D, Nangia V, Bron A, Tapply I (2024). Global estimates on the number of people blind or visually impaired by glaucoma: A meta-analysis from 2000 to 2020. Eye.

[5] Tham YC, Li X, Wong TY, Quigley HA, Aung T, Cheng CY (2014). Global prevalence of glaucoma and projections of glaucoma burden through 2040: a systematic review and meta-analysis. Ophthalmology.

[6] Kopilaš V, Kopilaš M (2024). Quality of life and mental health status of glaucoma patients. Front Med.

[7] Patel S, Pasquale LR (2010). Glaucoma drainage devices: a review of the past, present, and future. Semin Ophthalmol.

[8] Minckler DS, Francis BA, Hodapp EA, Jampel HD, Lin SC, Samples JR (2008). Aqueous shunts in glaucoma: a report by the American Academy of Ophthalmology. Ophthalmology.

[9] Gupta S, Jeria S (2022). A Review on Glaucoma Drainage Devices and its Complications. Cureus.

[10] Oatts JT, Han Y (2023). Glaucoma Drainage Device Implantation, Outcomes, and Complications. Int Ophthalmol Clin.

[11] Kim MS, Kim KN, Kim CS (2016). Changes in Corneal Endothelial Cell after Ahmed Glaucoma Valve Implantation and Trabeculectomy: 1-Year Follow-up. Korean J Ophthalmol.

[12] Iacubitchii M, Bendelic E, Paduca A, Cociug A, Fernandez MJG (2024). In vivo Evaluation of PMMA Antiglaucoma Shunt’s Biocompatibility. IFMBE Proceedings 6th Int Conf Nanotechnologies Biomed Eng.

[13] Rouse JM, Sarkisian SR (2012). Mini-drainage devices: the Ex-PRESS Mini-Glaucoma Device. Dev Ophthalmol.

[14] Khouri AS, Khan MN, Fechtner RD, Vold SD (2014). Technique for removal of malpositioned Ex-PRESS glaucoma device. J Glaucoma.

[15] Bendelic E, Alsaliem S (2021). Șunt antiglaucomatos cu supapă. 1493 Int.Cl.: A61F 9/007 (2006-01). Bul Of Propr Intelect.

[16] Bendelic E (2020). Raport anual privind implementarea proiectului din cadrul Programului de Stat (2020-2023). Implementarea unei metode chirurgicale în tratamentul glaucomului cu implantarea şuntului cu supapă elaborat.

[17] Huang Q, Cheng GPM, Chiu K, Wang GQ (2016). Surface modification of intraocular lenses. Chin Med J (Engl).

[18] Trocme SD, Li H (2000). Effect of heparin-surface-modified intraocular lenses on postoperative inflammation after phacoemulsification: A randomized trial in a United States patient population. Ophthalmology.

[19] Overby DR, Clark AF (2015). Animal models of glucocorticoid-induced glaucoma. Exp Eye Res.

[20] Hester DE, Trites PN, Peiffer RL, Petrow V (1987). Steroid-Induced Ocular Hypertension in the Rabbit: A Model Using Subconjunctival Injections. J Ocul Pharmacol.

[21] Khan SU, Ashraf M, Khan AS, Ali Z, Rahman MU, Shah A (2014). Steroid induced ocular hypertension: an animal model. Gomal J Med Sci.

[22] Iacubițchii M, Bendelic E, Paduca A, Magla T (2021). Surgical procedure of antiglaucomatous shunt with valve implantation in laboratory rabbit. Cercet în Biomed și sănătate calitate.

[23] Iacubitchii M, Paduca A, Suleiman A, Iacubitchii V (2020). Surgical treatment in induced ocular hypertension in rabbit. MedEspera Int Med Congr Students Young Dr. Abstract Book.

[24] Hau S, Bunce C, Barton K (2021). Corneal Endothelial Cell Loss after Baerveldt Glaucoma Implant Surgery. Ophthalmol Glaucoma.

[25] Ishida K, Nakano Y, Ojino K, Shimazawa M, Otsuka T, Inagaki S (2021). Evaluation of Bleb Characteristics after Trabeculectomy and Glaucoma Implant Surgery in the Rabbit. Ophthalmic Res.

[26] Bagnasco L, Bagnis A, Bonzano C (2020). Terminology and guidelines for glaucoma. European Glaucoma Society Terminology and Guidelines for Glaucoma.

[27] Dave B, Patel M, Suresh S, Ginjupalli M, Surya A, Albdour M, Kooner KS (2024). Wound Modulations in Glaucoma Surgery: A Systematic Review. Bioengineering (Basel).

[28] Parikh KS, Josyula A, Omiadze R, Ahn JY, Ha Y, Ensign LM, Hanes J, Pitha I (2020). Nano-structured glaucoma drainage implant safely and significantly reduces intraocular pressure in rabbits via post-operative outflow modulation. Sci Rep.

